# Distant metastasis dynamics following subsequent surgeries after primary breast cancer removal

**DOI:** 10.1186/s13058-019-1139-7

**Published:** 2019-05-02

**Authors:** Romano Demicheli, Hanna Dillekås, Oddbjørn Straume, Elia Biganzoli

**Affiliations:** 1 Laboratory of Medical Statistics, Biometry and Bioinformatics “Giulio A. Maccacaro”, Department of Clinical Sciences and Community Health, University of Milan Campus Cascina Rosa, Fondazione IRCCS Istituto Nazionale Tumori, via Vanzetti 5, 20133 Milan, Italy; 2 Department of Oncology, Haukeland University Hospital, N 5021 Bergen, Norway; 3 Department of Clinical Science, University of Bergen, N 5012 Bergen, Norway; 4 Centre of Cancer Biomarkers, University of Bergen, N 5012 Bergen, Norway; 5 Laboratory of Medical Statistics and Epidemiology, “Giulio A. Maccacaro”, Department of Clinical Sciences and Community Health, University of Milan, Milan, Italy

**Keywords:** Breast cancer, Recurrence dynamics, Metastasis development, Second surgery, Tumour homeostasis, Surgery-related metastasis acceleration

## Abstract

**Background:**

The aim of the research was to separate the distant metastasis (DM) enhancing effect due to breast tumour removal from that due to surgical manoeuvre by itself.

**Methods:**

DM dynamics following surgery for ipsilateral breast tumour recurrence (IBTR), contralateral breast cancer (CBC) and delayed reconstruction (REC), which was performed after the original breast cancer surgical removal, was analysed. A total of 338 patients with IBTR, 239 with CBC and 312 with REC were studied.

**Results:**

The DM dynamics following IBTR, CBC and REC, when assessed with time origin at their surgical treatment, is similar to the analogous pattern following primary tumour removal, with a first major peak at about 18 months and a second lower one at about 5 years from surgery. The time span between primary tumour removal and the second surgery is influential on DM risk levels for IBTR and CBC patients, not for REC patients.

**Conclusions:**

The role of breast tumour removal is different from the role of surgery by itself. Our findings suggest that the major effect of reconstructive surgery is microscopic metastasis acceleration, while breast tumour surgical removal (either primary or IBTR or CBC) involves both tumour homeostasis interruption and microscopic metastasis growth acceleration. The removal of a breast tumour would eliminate its homeostatic restrains on metastatic foci, thus allowing metastasis development, which, in turn, would be supported by the forwarding action of the mechanisms triggered by the surgical wounding.

## Background

In the middle of the nineteenth century, the arguments set out by Virchow, who suggested that the disease starts as a single focus within the breast, then migrates to the axillary lymph nodes and ultimately to distant organs, supported the Halsted operation that was adopted as the default therapy worldwide [1]. However, among resected patients, 30% of node-negative and 75% of node-positive women still developed distant metastases and succumbed [2]. The failure of mastectomy and other more aggressive operations to cure patients and, moreover, novel biology-based assumptions on the disease course [3] suggesting that the extent of local treatment does not affect survival supported a reduction of the extent of surgery. Additionally, clinical investigations and mathematical modelling advocated that surgical resection might not always be beneficial [4, 5] providing evidence that, while it favourably modifies the natural history of breast cancer for the majority of patients, it may also hasten the metastatic development for a number of them, by triggering growth of occult tumour deposits. The concepts underlying this new model extended to the clinical level the results of a protracted history of investigations lasting more than a century [6].

Helpful hints about the new model were achieved from analyses of post-resection recurrence dynamics in early breast cancer patients undergoing potentially curative removal of the primary tumour [7, 8]. A model assuming post-surgery acceleration of disease progression by a burst of growth in previously dormant micrometastases appeared to best fit the clinical data. Similar findings were observed in non-small cell lung cancer [9]. While this acceleration apparently occurs at the time of local treatment, it is still not deciphered whether this effect can be ascribed to primary tumour removal (e.g. to removal of inhibitory factors) or to the surgical manoeuvre per se (e.g. CTC release, immune suppression and pro-angiogenic stimulus of wounding) or to both. This differentiation is important as it may open a window to new therapeutic approaches.

Clues about this subject may be detected by the analysis of the recurrence dynamics in patients who undergo subsequent breast surgical manoeuvres during the follow-up of the disease in addition to primary tumour surgical removal. Patients undergoing conservative surgery for their primary tumour may experience ipsilateral breast tumour recurrence (IBTR), and others may be diagnosed contralateral breast cancer (CBC) whatever the surgical approach for the primary tumour has been. Moreover, some patients undergoing mastectomy as the first surgical treatment call for breast reconstruction (REC) and undergo aesthetical surgery. We hypothesized different metastatic recurrence dynamics associated with the different surgical procedures due to the clinical presence (IBTR and CBC) or absence (REC) of a tumour reservoir in the breast. Therefore, we report here findings from the analysis of distant metastasis (DM) dynamics following IBTR, CBC and REC, which was carried out with the aim of unravelling the different roles, if any, of the two possible factors, i.e. breast tumour removal and surgical manoeuvre by itself.

## Patients and methods

At the National Cancer Institute of Milan, three randomized clinical trials have been carried out in the past, investigating the role of different surgical approaches for primary tumour removal. Moreover, since preliminary results of the first trial on the breast-conserving treatment for early breast cancer, which provided evidence that conservative surgery plus chest wall radiotherapy was comparable to more aggressive resections [10], patients received breast-conserving treatment as routine practice outside randomized clinical trials (out-trial patients). All axillary node-positive (N+) patients were offered systemic adjuvant treatment with cyclophosphamide + methotrexate + fluorouracil (CMF) or CMF plus doxorubicin, while no further post-surgical systemic treatment was recommended to axillary node-negative (N−) patients. Adjuvant hormone therapy was not utilized within the randomized clinical trials and seldom employed for out-trial patients, as it was not considered mandatory at that time. Two other randomized clinical trials were accomplished on patients who, following mastectomy or breast-conserving treatment, were found to be axillary node positive (N+). Patients with one to three positive axillary lymph nodes were randomly allocated to receive either 12 courses of CMF or 8 courses of the same regimen followed by 4 courses of doxorubicin, while patients with > 3 positive axillary nodes were randomized to receive either four courses of doxorubicin followed by 8 courses of CMF or 2 courses of CMF and 1 course of doxorubicin for a total of 12 courses. All clinical data from patients enrolled into the reported clinical trials or treated outside of trials were systematically recorded and stored in standard format. Detailed descriptions of patients, treatments and follow-up modalities have been reported elsewhere [10–14]. In particular, data for patients suffering IBTR are reported in ref. [15].

A further database was analysed, including all breast cancer patients undergoing mastectomy who underwent delayed reconstructive surgery at Haukeland University Hospital, Bergen, Norway. The reconstructive procedures were implant surgery, implants combined with flaps, deep inferior epigastric perforator flaps and transverse rectus abdominis myo-cutaneous flaps. A paired control was randomly selected from patients with identical T and N stages, age ± 2 years, and follow-up without recurrence equal to or longer than the time to reconstruction of the respective matched reconstructed patient (defined as “reference time”, i.e. the time origin for the analysis of DM dynamics for controls). Patient characteristics and details of the study have been reported in ref. [16]. All studies supplying the analysed databases were approved by the institutional ethics committees and review boards in accordance with the Declaration of Helsinki.

**Fig. 1:**
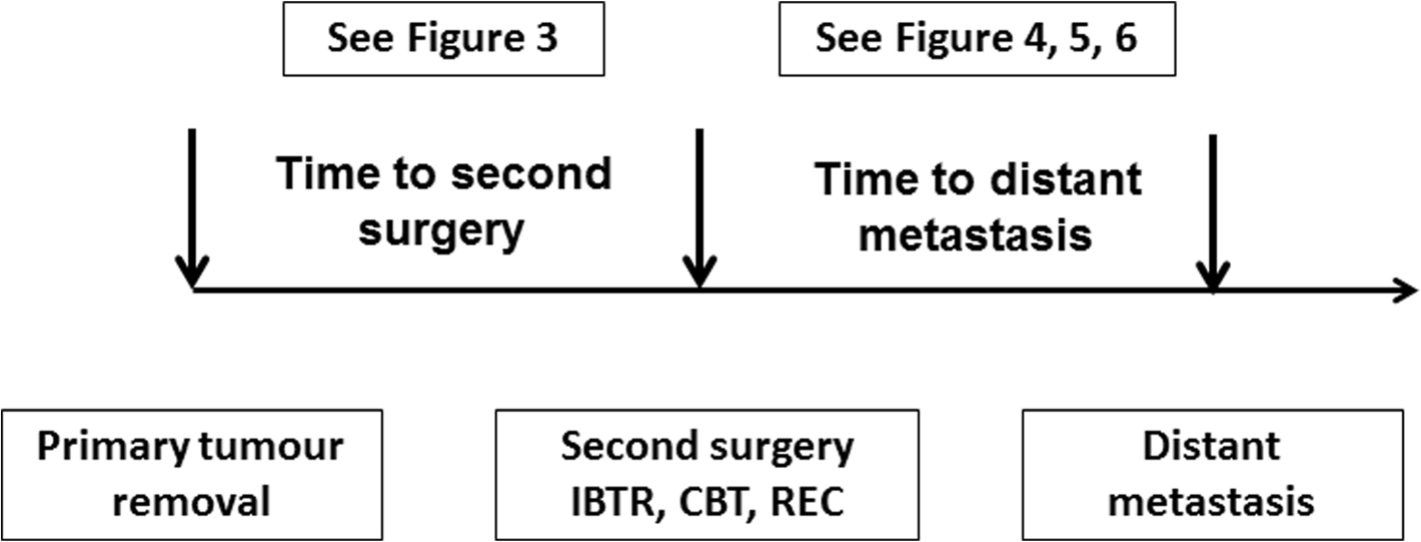
Analysed times: time to second surgery (from primary cancer surgery to surgery for ipsilateral breast tumour recurrence (IBTR), contralateral breast cancer (CBC) or breast reconstruction (REC)) and time to distant metastasis (from the second surgery to the appearance of distant metastasis)

The analysis of recurrence dynamics was focused on DM as the first event after the second surgery (the studied timing periods are outlined in Fig. 1): DM-free survival times were calculated as time elapsed since the second surgery (for IBTR, CBC or REC) to DM occurrence or to the last documented follow-up with no evidence of disease. Second primary tumours, including contralateral breast cancers, were considered as competing events, and the corresponding event-free survival times were censored at the time of their occurrence. The DM dynamics was studied by estimating with the life-table method the hazard rate for DM, i.e. the conditional probability of manifesting DM during a certain time span, given that the patient is clinically DM free at the beginning of the interval [17]. A discretization of the time axis in 6-month units was applied, and a Kernel-like smoothing procedure [18] was adopted.

## Results

**Fig. 2:**
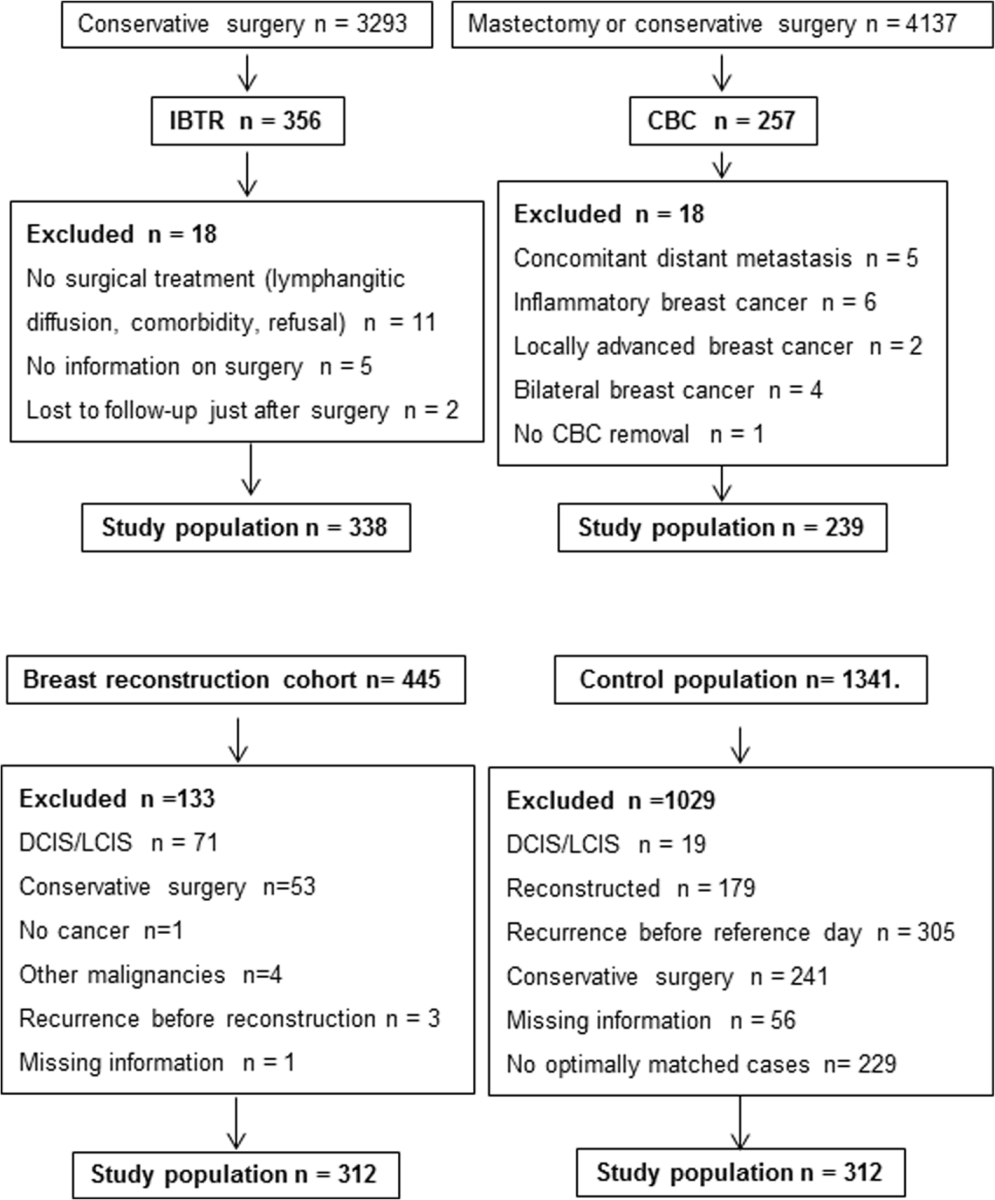
A CONSORT diagram for IBTR, CBC, REC and control patients. Analysed data were from trials carried out between 1975 and 1990 (IBTR, CBC) and between 1977 and 2007 (REC, controls)

A CONSORT diagram for IBTR, CBC and REC patients and matched controls is reported in Fig. 2.

Among patients undergoing conservative surgery, 92% received chest wall radiation therapy, mostly at the total dose of 50 Gy (daily dose 2 Gy) with high energy plus 10 Gy (daily dose 2 Gy) as a boost with orthovoltage to the ipsilateral breast. Following the diagnosis of IBTR or CBC, the treatment was decided on an individual basis. Median follow-up times after IBTR and after CBC were 151 months and 144 months, respectively. The number of patients suffering DM diagnosis within 10 years from IBTR and CBC was 138 and 84, respectively.

In the Norwegian study, the matched control group included patients who were extracted from a total of 868 (see the “Patients and Methods” section in ref. [16]). Median follow-up after reconstruction or reference time for controls was 137 months. The number of patients suffering DM within 10 years for REC and controls was 44 and 45, respectively.

Main patient characteristics at primary tumour treatment are reported in Table 1. In spite of the wide time span of patient accrual, the homogeneity of main prognostic factors across the databases, with the exception of tumour size in REC patients, is noteworthy. Axillary node involvement is near identical, as well as the frequency of ER-positive and ER-negative tumours among assessed ones, despite the fact that ER content was measured at different frequencies in the three series. Anyway, the DM dynamics of ER+ and ER− cancer have similar timing pattern [19] and, therefore, no modification of surgery effects on the time patterns was expected by oestrogen receptor levels. Moreover, as analysed patients suffering IBTR and CBC did not receive adjuvant endocrine therapy just like 42% of ER+ reconstructed patients, the question of whether endocrine therapy may alter DM patterns in a modern cohort remains open. HER2 status was not available and, accordingly, no specific treatment was administered.

**Table 1:** Patient characteristics

	IBTR (338)	CBC (239)	Rec (312)	Controls (312)
Median age at diagnosis (years)	45	48	48	49
25%–75%	39–52	42–56	42–53	43–53
Range	21–69	22–75	29–73	28–71
Tumour size (%)				
T1	85	84	61	62
T2	12	15	29	30
T3/4	–	–	8	7
Missing	3	1	2	1
Node negative (%)	70	64	68	67
Node positive (%)	30	36	32	33
ER negative (%)	16	16	20	19
ER positive (%)	63	53	70	70
ER missing (%)	21	31	10	11

**Fig. 3:**
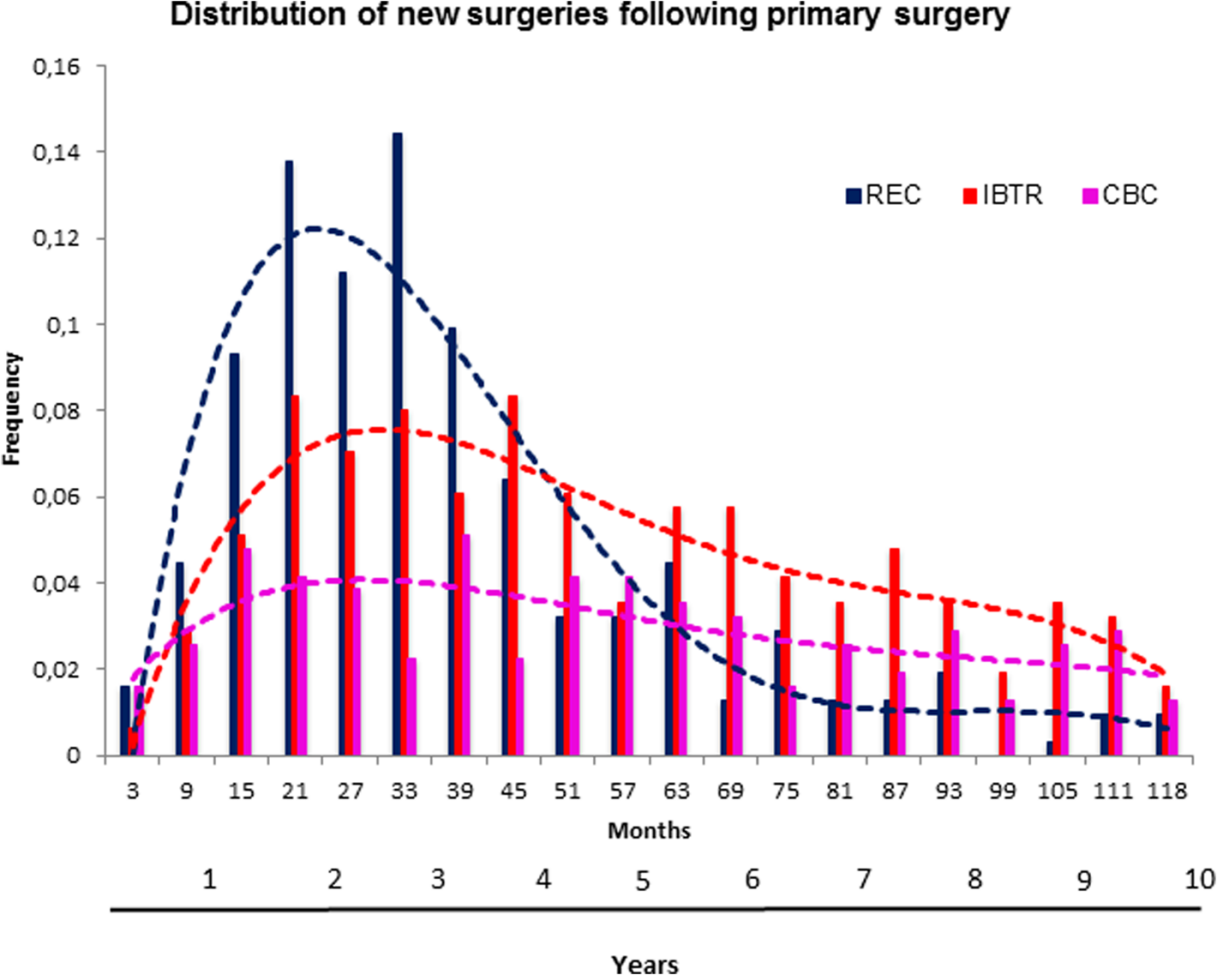
Distribution of surgical treatments for IBTR (red line), CBC (fuchsia line) and reconstruction (blue line) during the follow-up with *t* = 0 at primary tumour removal. RECs were performed mainly during the first 5 years (median time 2.5 years) while IBTR removal had a more protracted distribution (median time 4.3 years) and CBCs had a steadier pattern consistent with the notion that the occurrence of a CBC may be considered a random event

The distribution of surgical treatments for IBTR, CBC or REC during the follow-up subsequent to primary tumour removal is reported in Fig. 3. Reconstructions were performed mainly during the first 5 years (median time 2.5 years) while IBTR removal had a more protracted distribution (median time 4.3 years) and a structured pattern [20]. CBC treatments have a steadier pattern consistent with the notion that the occurrence of a CBC may be considered a random event not time-related with primary tumour [21, 22].

**Fig. 4:**
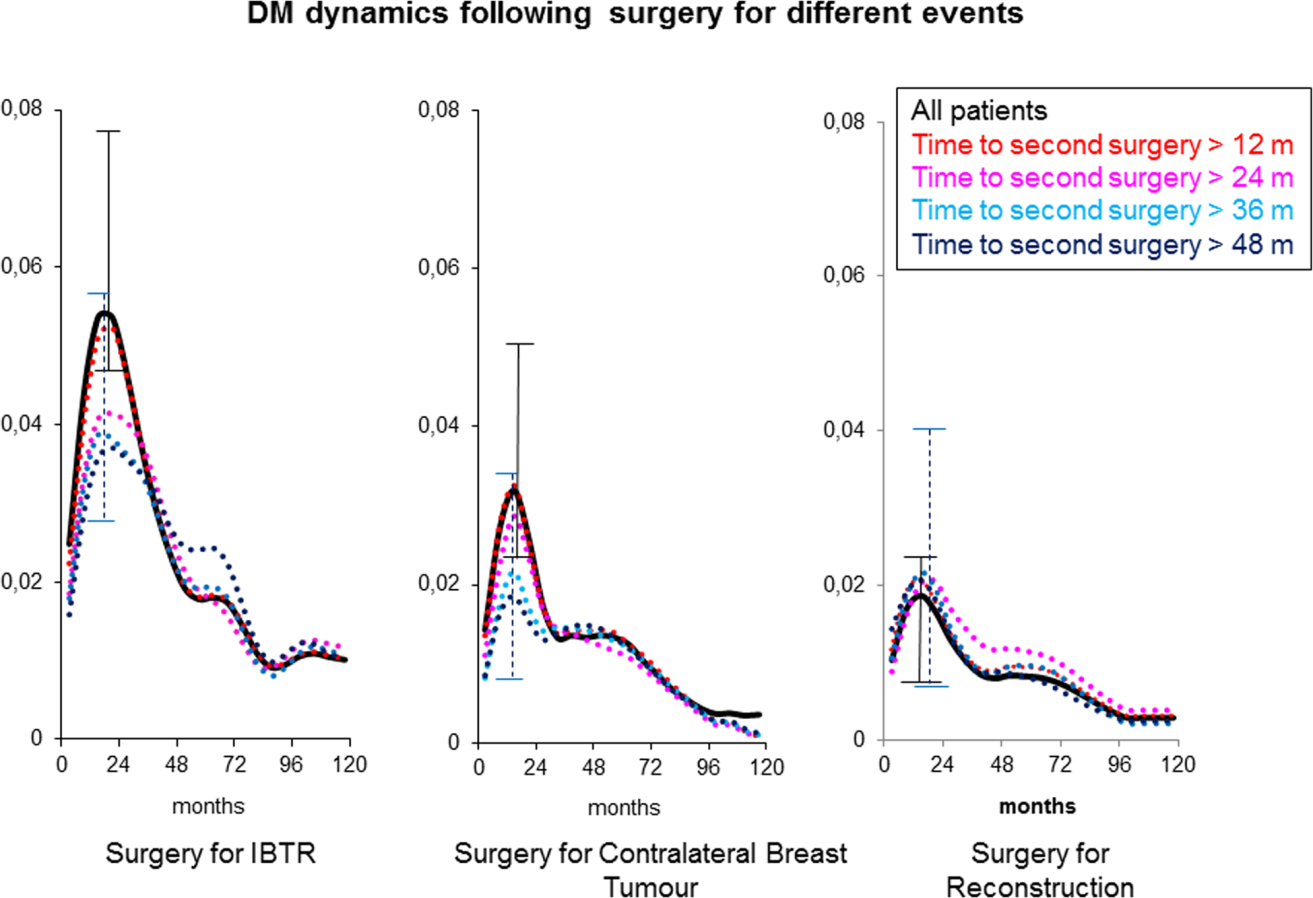
Distant metastasis dynamics following surgery for IBTR, contralateral breast tumour and reconstruction (*t* = 0 at second surgery). The hazard rate pattern is similar for the three groups with a first major peak at about 24 months and a second lower one at about 5 years. The analysis was performed for all patients (bold lines) and, moreover, by time from primary tumour removal to second surgery as well (dashed lines). While this factor is not influent for reconstructed patients, it changes the first peak height for surgeries removing neoplastic nodules if the time to second surgery is less than about 3 years. Confidence intervals on the estimated hazard rates at the first peak position are reported for all patients and those with time to second surgery more than 48. *Y*-axis reports hazard rates for DM, i.e. the conditional probability of manifesting DM during an interval of 6 months, given that the patient is clinically DM free at the beginning of the interval. *X*-axis units are months between primary tumour removal and the second surgical manoeuvre

**Fig. 5:**
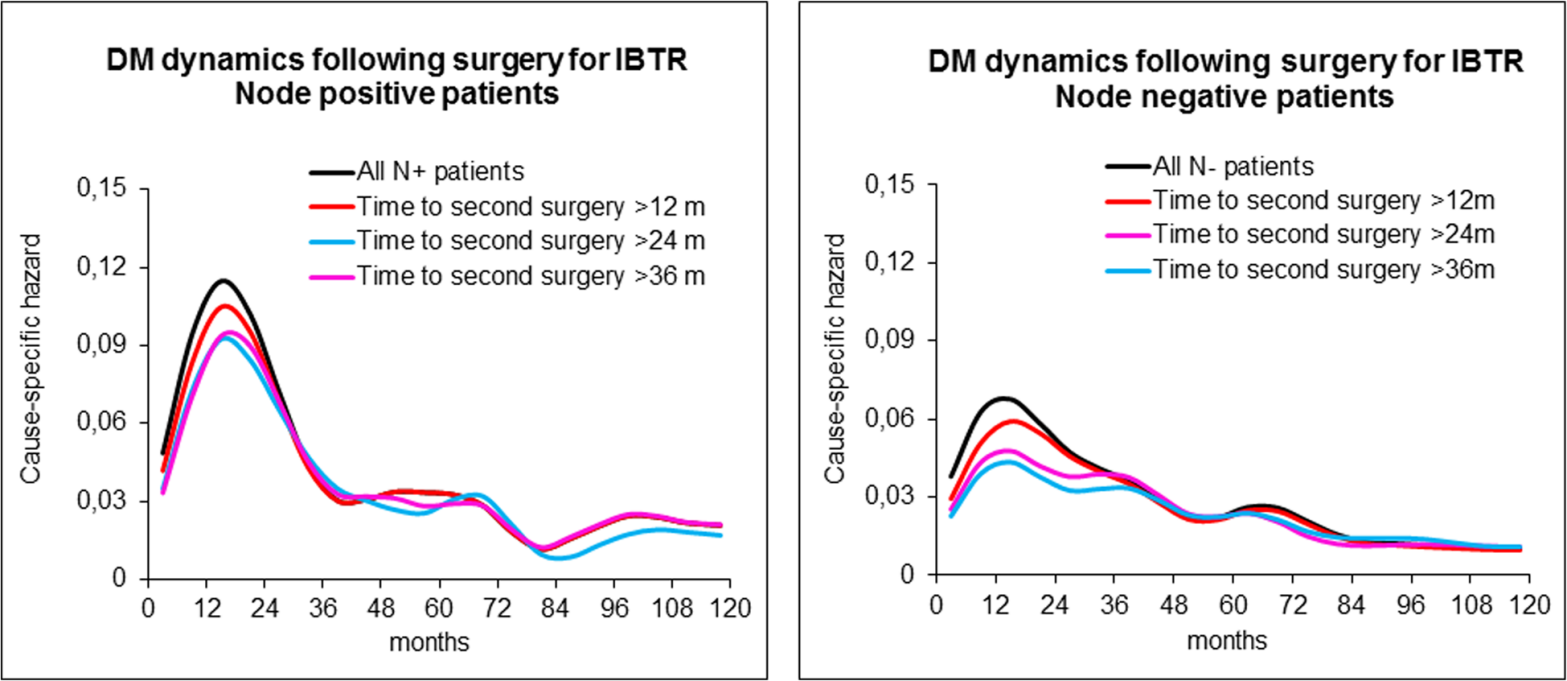
Distant metastasis dynamics following surgery for IBTR (*t* = 0 at IBTR) in patients with axillary node positive (left) or negative (right). The analysis was performed for all patients and, moreover, by time from primary tumour removal to surgery for IBTR. While nodal status affects the risk level, which is higher for node-positive patients, the time to surgery for IBTR is still influential on the first peak height if it is less than about 3 years. *Y*-axis reports hazard rates for DM, i.e. the conditional probability of manifesting DM during an interval of 6 months, given that the patient is clinically DM free at the beginning of the interval. *X*-axis units are months between primary tumour removal and the second surgical manoeuvre

**Fig. 6:**
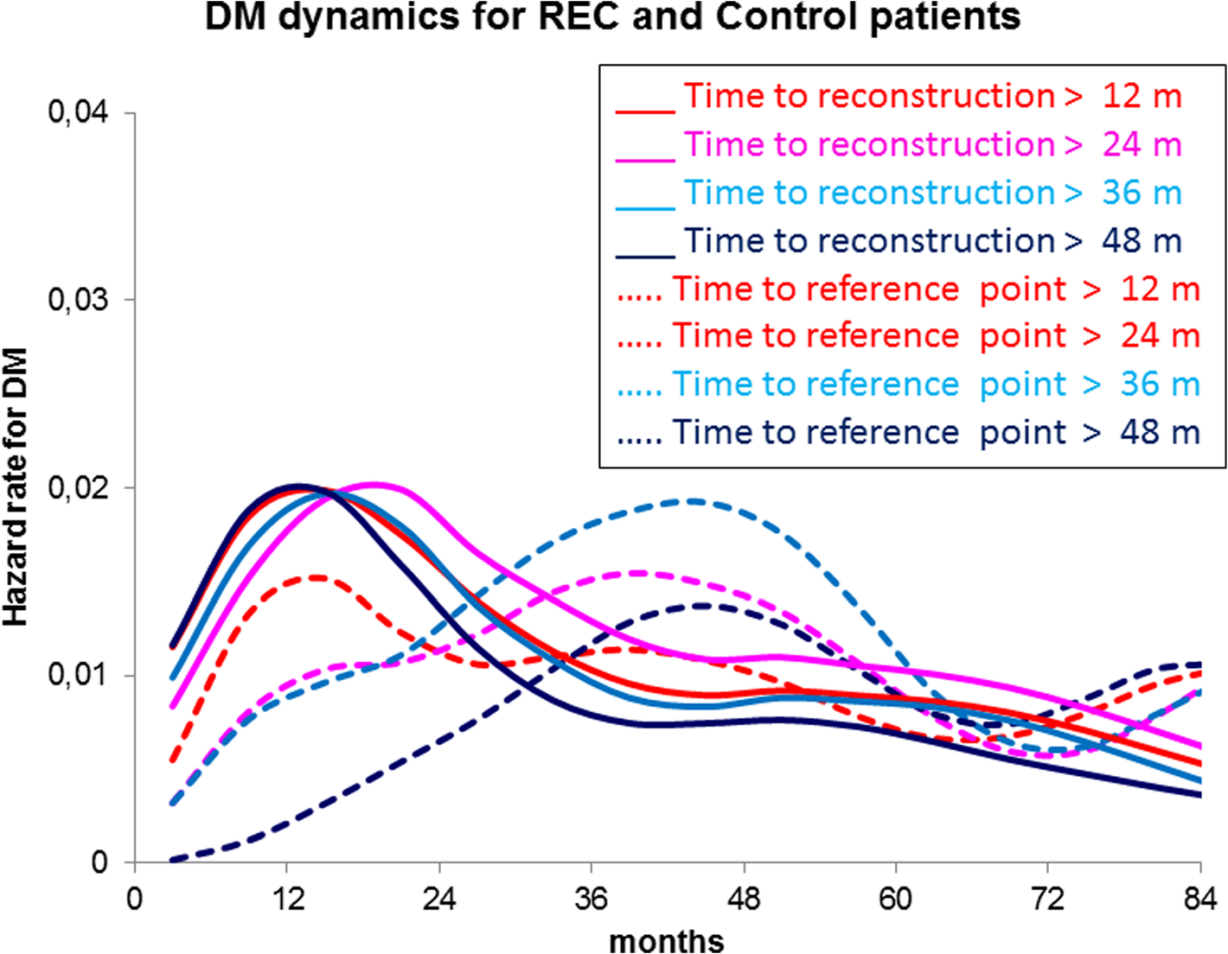
DM dynamics for reconstructed patients and paired controls. Following surgery for reconstruction (*t* = 0 at second surgery), the hazard rate for DM displays a common pattern that is not dependent on the time from primary tumour removal to second surgery. On the contrary, the analogous analysis for controls (*t* = 0 at reference time, as defined in the “Patients and methods” section) displays a progressive change of the DM dynamics that is compatible with the time-related DM risk expected after primary tumour removal in the absence of the further surgical manoeuvre. *Y*-axis units are hazard rates for DM, i.e. the conditional probability of manifesting DM during an interval of 6 months, given that the patient is clinically DM free at the beginning of the interval. *X*-axis units are months between primary tumour removal and the second surgical manoeuvre

The DM dynamics was analysed for the four groups in a timeframe with *t* = 0 at second surgery (Figs. 4 and 5 solid lines). Moreover, the influence of the time elapsed from primary tumour removal to the second surgical manoeuvre [time to second surgery] on the hazard rate for DM pattern was investigated as well (Figs. 4 and 5 dashed lines). The hazard rate pattern is similar for the three surgical groups with a first major peak at about 18 months and a second lower one at about 5 years from the second surgery, although the three levels of recurrence risk are different. Time to second surgery is apparently not influential for reconstructed patients, whereas it changes the first peak height for the other sets, showing that the influence is maximal for early re-operations, decreases afterwards and apparently disappears for time to second surgery values larger than 2 to 3 years. To ascertain whether factors known to be influent on the risk level may drive the described phenomenon, we analysed the DM dynamics by time to second surgery in IBTR patients pooled by axillary node status (node positive vs node negative) and by second surgery extent (mastectomy vs conservative surgery). In all analysed subsets, the time to second surgery aroused the same hazard rate pattern, as Fig. 5 exemplifies for the axillary nodal involvement. A comparison between REC patients and controls for DM hazard rate pattern is reported in Fig. 6, where the accelerating effect of surgery on the DM dynamics is quite evident.

## Discussion

Our analysis on breast cancer patients undergoing breast surgery for IBTR, CBC and REC provides two main results: (1) the DM dynamics following a new breast surgical manoeuvre performed after primary tumour removal is similar to the analogous dynamics following primary tumour removal and (2) the time span between the two operations is not associated with changes in peak timing; yet, for IBTR and CBC patients, it is related to progressive reduction of DM risk levels, while REC patients display similar DM risk levels for all time spans.

It should be emphasized that the three surgeries are performed in different clinical situations. Surgery for IBTR is strictly related to the specific multimodal dynamics of IBTR appearance [15] while CBC diagnosis and subsequent removal is an independent event with steady hazard rate [21, 22] and, finally, REC surgery is related to the patient’s desire and only indirectly to clinical conditions. Therefore, when comparative analyses among patients with such different tumour-host settings at the time of surgery display similar behaviours, they are reasonably attributable to the act of treatment per se. Our findings suggest that there is a metastasis-enhancing effect in all surgical interventions, which displays, however, different traits related to whether a macroscopic breast tumour is removed or not.

The enhancing effect of surgical primary tumour removal on metastatic disease is well supported by a long history of investigations [6] and from a few clinical studies in humans (e.g. [23]). It enables to explain the multipeak pattern of the hazard rate for recurrence in patients with early breast cancer undergoing surgery or surgery plus adjuvant chemotherapy [7]. Here, we confirm that this enhancing effect is observable even when surgical manoeuvres are performed in the breast area at a later date. The result of our analysis apparently diverges from the outcome of a previous investigation on the same subject [24] suggesting that traumas or intervening surgical procedures unrelated to cancer are not associated with an increased cumulative rate of breast cancer recurrence in a 2-year window. Although this discrepancy may be related to the shortness of the analysed interval, a subtler inference could be considered on the basis of the known topological specificity of cell populations [25, 26] and of the finding that, in an animal model, acceleration in tumour growth by mobilization of bone marrow-derived cells may be different after operative injuries to different organs [27]. Indeed, one could speculate that surgery in the breast area may stimulate distant breast cancer foci, unlike surgery in other sites. Unfortunately, this hypothesis cannot be disentangled since data on non-breast interventions were not available for comparison.

Of note, the hazard rate peaks, in particular the first one, have different heights in IBTR, CBC and REC (Fig. 4). This occurrence is in keeping with the notion that the sudden acceleration of metastasis development takes action on the underlying DM dynamics, which is different in IBTR, CBC and REC patients. Indeed, patients with IBTR have an intrinsic high risk of DM (similar to N+ patients) that was unpredictable by the usual prognostic factors at the initial treatment and that is revealed when IBTR emerges in advance of the competing DM events [15, 28]. In comparison with these patients, women suffering CBC display a considerably lower first hazard rate peak (about 60% peak to peak) in keeping with the concept that CBC is a second primary, unrelated to the first one [21, 29, 30]. Accordingly, patients with CBC actually fit to a population with “average” DM risk [31]. Finally, patients undergoing REC have better prognosis due to favourable selection criteria: they had no previous recurrence event and factors such as smoking, obesity and diabetes excluded patients from being offered complex breast reconstructive procedures. Moreover, the baseline risk in this population may be influenced by features here not accounted for, such as socioeconomic conditions, better general health and low body mass index, which is recently emerging as a prognostic factor in breast cancer [32].

While there is a substantial body of evidence indicating that the surgery-associated tissue trauma and wound healing can promote growth, angiogenesis and metastatic ability of cancers [33], data on the possible homeostatic connection between primary tumour and its metastases are lacking. Our finding that the time to second surgery is influential on the risk level only for IBTR and CBC patients and not for REC patients suggests that tumour removal, which occurs in the former groups only, plays a specific role on metastasis development. This idea is in agreement with the recalled model of breast cancer metastatic development [7] if one takes into account that the manifestation of IBTR or CBC, i.e. of a macroscopic breast tumour, is preceded by a number of months of subclinical disease. According to the tumour homeostasis concept [7], during this time span, the growing tumour exerts constraints on distant microscopic foci, somehow mimicking the homeostatic processes underlying the control of size in adult organs and organisms [34, 35]. Although the molecular characteristics of these mechanisms are largely unknown, recent reports have provided initial interesting findings [36, 37], which may have oncological important implications as well [38].

In patients suffering IBTR or CBC, a number of metastatic foci are related to the previous breast cancer, although a few of them may be associated with the new breast neoplastic lump. The emerging restrictive interference results into some freeze of the microscopic metastases in the conditions existing when the new homeostatic action is starting. Taking into account the hazard rate dynamics for the DM related to the primary breast cancer [7], such a freeze should have effects depending on time to second surgery: the shorter this time, the higher the underlying DM risk. Consequently, while the DM dynamics after the IBTR or CBC removal maintains the usual time-related pattern, the corresponding hazard rate level would depend on time to second surgery. Following 2 to 3 years, the time to second surgery loses its prognostic value, in keeping with the drop of DM risk attributable to the primary breast cancer [7].

The finding that patients undergoing REC do not present any effect from the time to second surgery, while displaying the usual time-related pattern in the post-reconstruction DM dynamics, suggests that the reconstructive surgical manoeuvre, in the absence of any breast tumour removal, may act on metastasis development differently from IBRT and CBC surgical removal. As a working hypothesis, it may be assumed that surgical manoeuvres prominently act on the microenvironment of tumour foci turning it into conducive (e.g. by activating angiogenesis) and thus sustaining growth [39]. This facilitating action would simply speed up the clinical appearance of some metastases that would emerge later according to their own dynamics. This hypothesis is suggested by the comparison between the hazard rate patterns for DM in REC patients and in the matched paired control group (Fig. 6). This comparison suggests that the reconstruction is associated with a decrease of the hazard rate for DM at the fourth year and a concomitant increase of the hazard rate at the second year.

The present findings are coherent with and integrate the evidence coming from separate studies on IBTR and REC, resorting to advanced time scale statistical modelling [16, 40]. The overall picture provided support to the biological hypothesis, underlying the observed distant metastasis dynamics following surgeries performed after primary breast cancer surgical removal, according to different tumour homeostasis-related and surgical wound-related effects on metastasis development.

The analysed databases did not included data on HER2, preventing investigations on triple-negative patients. Moreover, we could not analyse the possible role of anaesthetic management, which potentially influences the long-term outcome most probably in patients undergoing more extended surgery [41], due to missing information about this factor.

## Conclusions

In summary, the findings of the present analysis support the concept that the impact of breast tumour surgical removal (either primary or IBTR or CBC) on microscopic metastases is twofold, inasmuch as two different factors, i.e. tumour homeostasis interruption and surgical wound effects, are involved. The removal of a breast tumour would result into the sudden elimination of the restrains on metastatic foci, thus allowing metastasis development, which, in turn, would be supported by the forwarding action of the mechanisms triggered by the surgical wounding. This surgery-related phenomenon would underlie the behaviour of DMs in the REC group, where no detectable tumour deposit is removed. While associations of such latter phenomenon with surgical-related inflammatory conditions and different anaesthesia modalities are suggested from a few clinical data [41–43], the biological mechanisms underlying tumour homeostasis are largely unknown. Investigations in this field are urgently warranted.

## Abbreviations

CBC: Contralateral breast cancer
CMF: Cyclophosphamide + methotrexate + fluorouracil
DM: Distant metastasis
IBTR: Ipsilateral breast tumour recurrence
N−: Axillary node negative
N+: Axillary node positive
REC: Delayed reconstruction
